# Actual Management of Anterior Calyceal Diverticular Calculi: A Challenging Flexible Retrograde Endoscopic Approach

**DOI:** 10.1155/2020/7983242

**Published:** 2020-09-16

**Authors:** Ali Barki, Anouar El Moudane

**Affiliations:** Department of Urology, Mohammed IV University Medical Center Oujda, Morocco

## Abstract

Calyceal diverticula is a cavity that communicates with the collecting system through a narrow isthmus of the kidney. The incidence of the formation of stones in calyceal diverticula is 10-50%. This paper reports three cases of two females and one male who presented with calyceal diverticular calculi; the patients have been, arbitrarily, selected between August and February 2019 at the urology department of our university hospital. A minimally invasive treatment includes extracorporeal lithotripsy (ESWL), and F-URS (flexible ureteroscopy) was performed. We report this case series.

## 1. Introduction

Calyceal diverticula constitute a cavity, which communicates with the collecting system through a narrow isthmus of the kidney. 0.21-0.45% of routine intravenous studies showed calyceal diverticula, which makes them uncommon [1]. Calyceal diverticula are usually asymptomatic but can eventually cause infection and become painful [2]. The incidence of stone formation in calyceal diverticula is 10-50% [3].

Asymptomatic calyceal diverticular calculi with no signs of infection can be managed just with surveillance. In other cases, the choice of the treatment will depend on the location of the calyceal diverticular calculi.

We report three different cases of two females and one male, who presented calyceal diverticular calculus treated with (F-URS) after the failure of ESWL; we performed a review of literature about the management of this kind of stones.

## 2. Case 1

A 61-year-old woman, with an unremarkable medical history, presented with bilateral lumbar pain evolving for two years. Physical examination was unremarkable, with no fever; the blood pressure was 120/70 mmHg. The laboratory test results were within the normal range with a serum creatinine level at 8.5 mg/l and a white blood cell count at 7.000 per microliter.

CT scan with contrast demonstrated three stone-bearing diverticula, in the middle calyces of the left kidney measuring respectfully 5, 6, and 8 mm, whereas the right kidney was damaged on long-standing obstruction.

**Figure 1:**
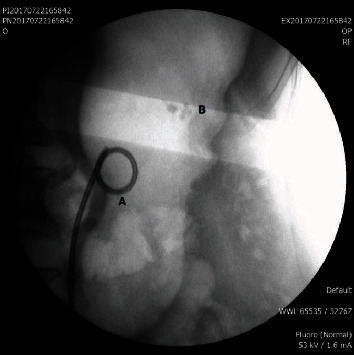
A: double pigtail stent (CH6) placed to dilate the ureter. B: 3 stone-bearing diverticula in middle calyces.

**Figure 2:**
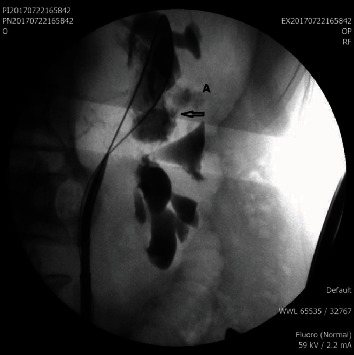
A: stone-containing calyceal diverticula. Arrow: opacified neck of a calyceal diverticulum.

**Figure 3:**
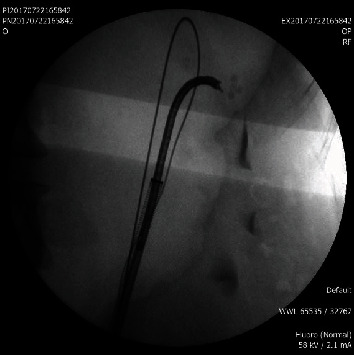
Pulverized calculi with the holmium laser.

ESWL was performed first; radiographic control did not show any progress. A double pigtail stent (CH6) was placed to dilate the ureter 3 weeks preoperatively (Figure 1). The retrograde pyelogram showed the stone contained in the calyceal diverticula with its neck opacified (Figure 2). The infundibulum of the caliceal diverticulum was incised with a calculase II holmium laser (230 microoptical fiber, 1.0 J-10 Hz), and the calculi were pulverized with low energy and high frequency (0.8 J-25 Hz) (Figure 3). The evolution was marked by the disappearance of symptoms, with no residual at a three-month follow-up.

## 3. Case 2

A 68-year-old woman, with no medical history, presented with right lumbar pain since one year ago with intermittent hematuria. Physical examination was unremarkable with no fever; the blood pressure was 130/70 mmHg. The laboratory test results were within the normal range with a serum creatinine level at 7 mg/l and a white blood cell count at 8.000 per microliter.

**Figure 4:**
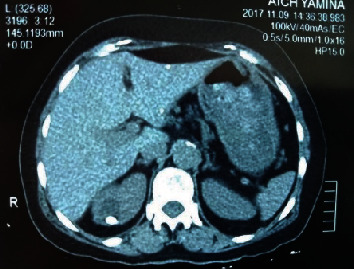
CT demonstrates a 10 mm stone-bearing diverticulum with 750 UH of density situated in the pole with the holmium laser.

CT scan with contrast demonstrated a 10 mm stone-bearing diverticulum with 750 UH of density situated in the upper pole (Figure 4).

**Figure 5:**
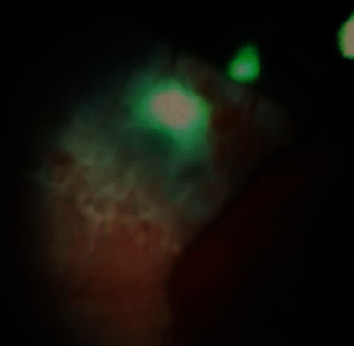
(F-URS) the infundibulum of diverticulum the calyceal diverticulum incised upper.

**Figure 6:**
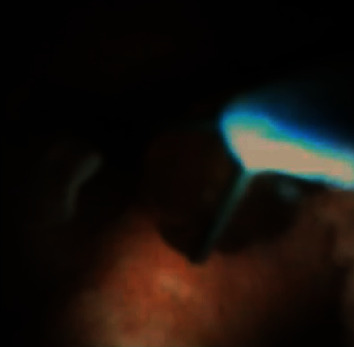
A fragment of calculi removed with a small caliber basket.

A double pigtail stent (CH6) was placed to dilate the ureter 3 weeks preoperatively. The retrograde pyelogram showed the stones contained in the calyceal diverticula, with its neck opacified. The infundibulum of the caliceal diverticulum was incised with a calculase II holmium laser (230 *μ*m optical fiber) (Figure 5), and the calculi were fragmented, with high energy and low frequency (1.2 J-6 Hz) into gravel less than 2 mm, and the rest of fragments were removed with a small caliber basket (Figure 6). In order to ensure that the kidney drains urine well after F-URS, a ureteral stent was left in place and then removed the following morning. The evolution was marked by the disappearance of symptoms, with no residual calculi at a three-month follow-up.

## 4. Case 3

A 57-year-old man, with an unremarkable medical history, presented with recurrent bilateral lumbar pain evolving for three years. Physical examination was unremarkable, with no fever; the blood pressure was 120/70 mmHg. The laboratory test results were within the normal range, with a serum creatinine level at 9.84 mg/l and a white blood cell count at 5020 per microliter.

CT scan with contrast and with 3D reconstruction demonstrated a 9.8 mm stone-bearing diverticulum with 1200 UH of density, located in both the anterior and middle calyces.

**Figure 7:**
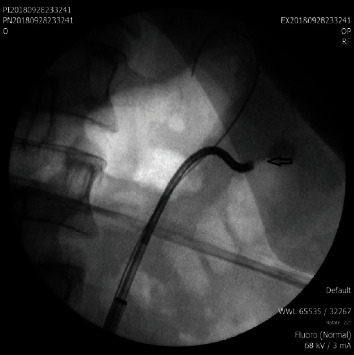
Arrow: opacified neck of a calyceal diverticulum.

**Figure 8:**
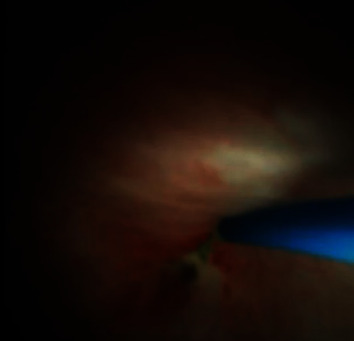
With F-URS, the infundibulum of the calyceal diverticulum is incised with the holmium laser.

A double pigtail stent (CH6) was placed to dilate the ureter for 4 weeks. The retrograde pyelogram showed stone-containing calyceal diverticula with an opacified neck of the calyceal diverticulum (Figures 7 and 8). The infundibulum of the caliceal diverticulum was incised with a calculase II holmium laser (230 *μ*m optical fiber), and the calculi were fragmented, with high energy and low frequency (1.2 J-6 Hz) into gravel less than 2 mm, and the rest of the fragments were removed with a small caliber basket. In order to ensure that the kidney drains urine well after F-URS, a ureteral stent was left in place and then removed the following morning. The evolution was marked by the disappearance of symptoms with no residual calculi at a three-month follow-up.

The follow-up of all patients at six months was uneventful.

## 5. Discussion

The congenital abnormality caused by the failure of regression of the ureteric buds leads rarely to calyceal diverticula, and it occurs equally with the same frequency in both sexes [4].

Calyceal diverticula are in general asymptomatic before stone formation [4, 5]. Asymptomatic calyceal diverticular calculi with no signs of infection can be managed just with surveillance: once the patient becomes symptomatic with the occurrence of hematuria and flank pain due to stone formation or development of recurrent urinary tract infection [6], several options of treatment can be used to manage symptomatic calyceal diverticular calculi. However, the choice of the treatment is established based on the diverticular location and size, the presence or absence of evident infundibulum, and the quality of overlying parenchyma.

The principal minimally invasive modalities, for managing calyceal diverticular calculi, are ESWL, PCNL (percutaneous nephrolithotomy), F-URS, and laparoscopy [2].

### 5.1. Extracorporeal Lithotripsy (ESWL)

The first choice of treatment can be the extracorporeal lithotripsy (ESWL) for all diverticular calculi whatever their location in the kidney, but a narrow diverticular neck blocks the free passage of stone fragments. It is difficult to demonstrate how adequate the fragmentation of stone is in calyceal diverticulum or dilated calyx for patients treated with ESWL [4]. ESWL can reach stone-free rates of only 4-20% and gives symptomatic amelioration in 36-70% of patients [7, 8]. Patients with small-sized superior calyceal diverticular calculi treated with ESWL present a high stone-free rate.

Streem and Yost suggested that the stone-free rate can be higher if ESWL was limited to the patients with small calculi (<1.5 cm) and evident infundibulum [9, 10]. Treating stone-bearing calyceal diverticula with ESWL should be practiced with attention even with these reports of admissible stone-free rates and amelioration of symptoms [9]. Jones et al. proved that even if initially patients are symptom-free, afterward they will need a retreatment [7].

### 5.2. Percutaneous Nephrolithotomy (PCNL)

The percutaneous approach has been used in diverse small series. The management of the calyceal diverticulum by the percutaneous route is challenging because the cavity is usually small and the identification of the diverticular neck is often difficult [9]. It is recommended to puncture the calyceal diverticulum directly [11]. The percutaneous approach is considered to be the most minimally invasive approach used in treating calyceal diverticula [4].

In the literature, the rate of stone-free in PCNL treatment for stone-bearing calyceal diverticula is about 70-100% [12]. Despite the good results of the PCNL approach, the risk postoperative of recurrence case of calyceal diverticula and residual calculi is not insignificant [1, 2, 4–11, 13], using PCNL which increases the risk of complications as severe hemorrhage, damage to the surrounding organs or the kidney parenchyma, sepsis, or even death [2]. The anterior location of the diverticulum can cause a difficult puncture, so the anatomical position of the diverticulum is major [14].

This approach allows fulguration and destruction of the walls of the diverticulum; the other techniques do not allow it [15]. If the diverticulum is in a superior and anterior calyx, the flexible ureterorenoscopy is recommended.

### 5.3. Flexible Ureteroscopy (F-URS)

Ureterorenoscopic (URS) management of diverticular stones is more effective than SWL monotherapy and can allow avoiding the complications and discomfort of the more invasive therapies like percutaneous or laparoscopic techniques.

With the advancement of the flexible ureterorenoscopy, and the appliance of the holmium laser energy, this technique can be suitable for the treatment of calyceal diverticulum. The rates of success and long-term symptom-free are higher with the F-URS approach. Thus, the F-URS approach has more advantages with a short duration of hospitalization and low risk of complications [16, 17].

Flexible ureteroscopy can be used for every location of the diverticulum but with a small burden with a short accessible diverticular neck. The significant problems that the urologist can face when using F-URS are maintaining adequate deflection and identification of the diverticular neck.

### 5.4. Laparoscopy and Retroperitoneoscopy

In 1994, Ruckle and Segura described the laparoscopic management of a stone-filled calyceal diverticulum [18]. Laparoscopic surgery is reserved for cases with large stones in anteriorly located diverticula, with a narrow neck and complex branched calculi, with thin overlying parenchyma [11]. Compared to ESWL, percutaneous nephrolithotomy and ureteroscopic management of laparoscopic surgery are considered to be the most invasive approach. This should be the last choice, only when the other options are not feasible.

## 6. Conclusion

The F-URS with the holmium laser is an effective option to manage stone-bearing calyceal diverticula mainly for the upper and mid, anterior, or posterior diverticula. This technique proved that F-URS is effective with a high rate of success and symptom-free, but we need many series to compare it with the other techniques.
